# Printable enzyme-embedded materials for methane to methanol conversion

**DOI:** 10.1038/ncomms11900

**Published:** 2016-06-15

**Authors:** Craig D. Blanchette, Jennifer M. Knipe, Joshuah K. Stolaroff, Joshua R. DeOtte, James S. Oakdale, Amitesh Maiti, Jeremy M. Lenhardt, Sarah Sirajuddin, Amy C. Rosenzweig, Sarah E. Baker

**Affiliations:** 1 Lawrence Livermore National Laboratory, 7000 East Avenue, Livermore, California 94551, USA; 2 Department of Molecular Biosciences and of Chemistry, Northwestern University, Evanston, Illinois 60208, USA

## Abstract

An industrial process for the selective activation of methane under mild conditions would be highly valuable for controlling emissions to the environment and for utilizing vast new sources of natural gas. The only selective catalysts for methane activation and conversion to methanol under mild conditions are methane monooxygenases (MMOs) found in methanotrophic bacteria; however, these enzymes are not amenable to standard enzyme immobilization approaches. Using particulate methane monooxygenase (pMMO), we create a biocatalytic polymer material that converts methane to methanol. We demonstrate embedding the material within a silicone lattice to create mechanically robust, gas-permeable membranes, and direct printing of micron-scale structures with controlled geometry. Remarkably, the enzymes retain up to 100% activity in the polymer construct. The printed enzyme-embedded polymer motif is highly flexible for future development and should be useful in a wide range of applications, especially those involving gas–liquid reactions.

## Abstract (web summary)

There is a need for small-scale reactors that convert methane emissions to more valuable products to reduce climate impacts. Here, the authors show that printing 3D structures of the pMMO enzyme enables continuous methane conversion under ambient conditions and reduces mass transfer limitations.

## Graphical abstract

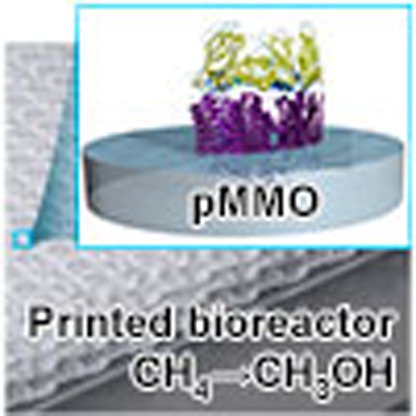

Advances in oil and gas extraction techniques have made vast new stores of natural gas (composed primarily of methane) available for use. However, substantial quantities of methane are leaked, vented or flared during these operations. Meanwhile, methane emissions contribute ∼1/3 of current net global warming potential, primarily from these and other distributed sources such as agriculture and landfills^1^. Compared with other hydrocarbons, and especially compared with the oil that is co-produced in hydrofracturing operations, methane has a much lower market value^2^ because of difficulty in methane storage and transport as well as its limited use as a transportation fuel. Current industrial technologies to convert methane to more valuable products, such as steam reformation followed by the Fischer–Tropsch process, operate at high temperature and pressure, require a large number of unit operations and yield a range of products^3^. Consequently, current industrial technologies have a low efficiency of methane conversion to final products and can only operate economically at very large scales^4^. A technology to efficiently convert methane to other hydrocarbons is highly sought-after as a profitable way to convert ‘stranded' sources of methane and natural gas (sources that are small, temporary or not close to a pipeline) to liquids for further processing.

The only known true catalyst (industrial or biological) to convert methane to methanol under ambient conditions with 100% selectivity is the enzyme methane monooxygenase (MMO)^5^,^6^, which converts methane to methanol (equation (1)) in methanotrophic bacteria.


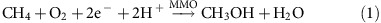


Partial methane oxidation by MMO enzymes can be carried out using engineered methanotroph organisms; however, this approach inevitably requires energy for upkeep and metabolism of the organisms, which reduces conversion efficiency. Engineered organisms that carry out more efficient methane activation have been proposed to overcome some of these challenges^4^. An additional challenge to whole-cell biocatalysis is that the reactions are typically carried out in low-throughput unit operations with mass transfer limitations, such as stirred tank reactors^4^,^7^. These mass-transfer limitations have been identified as a major hurdle to economical bioconversion^4^. A promising hybrid industrial–biological approach that could address the limitations with whole-cell biocatalysis is to separate the MMO enzyme from the host organism. Isolated enzymes offer the promise of highly controlled reactions at ambient conditions with higher conversion efficiency and greater flexibility of reactor and process design^8^.

There are both soluble (sMMO) and particulate (pMMO) forms of MMO^5^,^9^. The use of pMMO is particularly attractive for industrial applications because pMMO comprises an estimated 80% of proteins in the cell membrane^10^,^11^,^12^. Thus, concentrating pMMO to a reasonable purity requires only isolating the membrane fraction of the lysed cells using centrifugation. In order to use pMMO most effectively, the traditional methods of enzyme immobilization and exposure to reactants are not sufficient. These typical methods include crosslinking enzymes or immobilizing them on a solid support so that they can be separated from the products^13^ and carrying out batch reactions in the aqueous phase in a stirred tank reactor. Enzyme immobilization allows the reuse of costly enzymes and is required for a continuous flow-based bioreactor process. However, pMMO is not amenable to standard immobilization techniques designed for soluble proteins because surfactant solubilization of purified pMMO can lead to a pronounced reduction in activity^14^,^15^,^16^,^17^. For soluble enzymes, high-surface-area porous inorganic supports^18^,^19^,^20^ have been extensively implemented for immobilization, and have been shown to enhance enzyme stability while achieving high enzyme loading in nanometer-scale pores. However, the majority of the surface area in mesoporous materials is accessible only to proteins significantly smaller than 50 nm (ref. 21), and would therefore be inaccessible to the large (>>100 nm), optically opaque vesicles and liposomes that comprise membrane-bound pMMO.

While the operation of a stirred tank reactor is relatively simple at any scale, it has several drawbacks, including low productivity, high operating costs and variability in the quality of the product^22^. Moreover, the stirred tank reactor is not the optimal design for gas to liquid reactions such as methane to methanol conversion since it does not allow efficient delivery of reactant gases to enzymes or organisms in the bulk solution. Gas delivery is often achieved by bubbling the gas through the liquid; however, this approach suffers from mass-transfer limitations. Furthermore, methane and oxygen are only sparingly soluble in aqueous solvents: 1.5 and 1.3 mM atm^−1^, respectively, at 25 °C (ref. 23). Reactant concentrations are necessarily solubility-limited when the enzymes or organisms are dispersed in the aqueous phase.

Given that methanotrophic bacteria have evolved to use methane as their sole source of carbon and energy, a more appropriate and effective approach in designing bioreactor components might emulate their method of immobilizing pMMO. In methanotrophs, pMMO is expressed abundantly in high surface area, folded lipid membrane structures that can comprise a large part of the cellular volume^15^. Although weakly soluble in water, methane and oxygen are much more soluble in lipids^24^ and some hydrophobic polymers, such as silicones^25^,^26^. The expression of pMMO in the lipid membrane is likely functionally significant: methane is ∼10 times more soluble in the lipid membrane than in the aqueous cytosol^24^. Whole-cell kinetic data suggest that pMMO activity is dependent on oxygen concentrations^27^; its dependence on methane concentrations is not established. Embedding enzymes that utilize gas phase reactants within an organic, polymeric material allows tuning of the gas solubility, permeability and surface area. Polymers are also readily utilized as feedstocks for various forms of three-dimensional (3D) printing, which offers the ability to rapidly prototype structures, and tune material architecture for the system configuration and mass transfer, heat and diffusion limitations.

Here we report several key advances toward implementing a pMMO-based biocatalytic process for selective methane conversion. We developed and optimized a biocatalytic material consisting of active pMMO embedded in polyethylene glycol diacrylate (PEGDA) hydrogel. PEGDA was selected as the primary polymer substrate because of its biocompatibility and ease of crosslinking under mild conditions. We used this material to build an initial flow-through reactor using pMMO, and further demonstrate that it can be used as a substrate for 3D printing. These findings have major implications for bioreactor design and industrial methane conversion.

## Results

### pMMO activity in PEG hydrogel

In an effort to develop a biocatalytic material that can be molded into controlled, predetermined structures with tunable permeability and surface area, we explored several methods for embedding *Methylococcus capsulatus* (Bath) pMMO in a PEGDA-based polymer hydrogel. Polymer supports are more flexible for immobilizing heterogeneous and larger biocatalysts, and have even been used to entrap whole cells^28^ for biocatalysis. In addition, polymer supports have highly tunable mechanical and chemical properties, and have been used for a wide range of applications including maintaining mechanical stability of immobilized enzymes in stirred tank reactors^29^, immobilizing enzymes for non-aqueous biotransformations^30^ and functioning as substrate-permeable materials for functional synthetic metabolic pathways^31^.

Our initial efforts focused on solubilizing the membrane preparations using surfactant so that the material could be incorporated homogeneously in the polymer. We found that any treatment of the membrane preparations with surfactant, including encapsulation in nanolipoprotein particles, led to a pronounced decrease in activity. This behaviour has been reported previously^11^,^17^ and possible explanations include surfactant interference with activity, surfactant interference with the cofactor or disruption of the protein structure^16^. However, mixing the membrane fractions, either as prepared or extruded as liposomes directly with low concentrations of PEGDA 575, gave promising results. Therefore, we focused on optimizing the activity and protein retention of membrane preparations with PEGDA 575.

**Figure 1:**
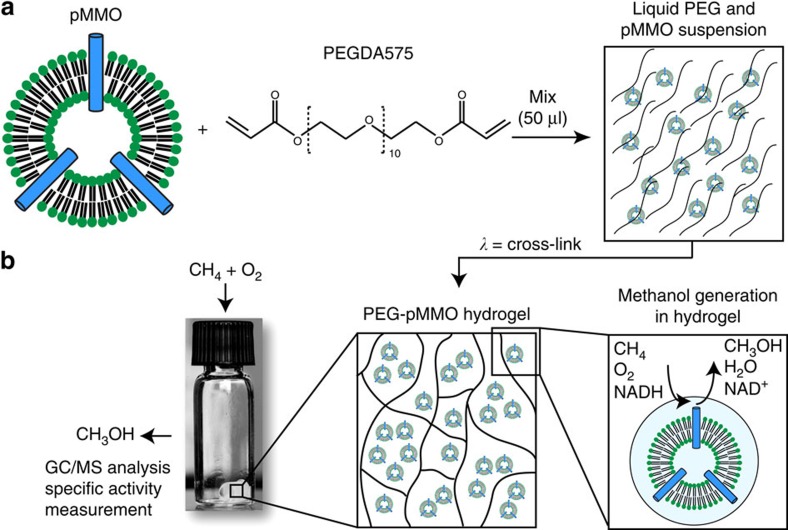
Schematic of pMMO encapsulation in hydrogel and activity assay. (**a**) Schematic of PEG-pMMO hydrogel fabrication. Membrane-bound pMMO is mixed with PEGDA 575 and photoinitiator and exposed to ultraviolet light to crosslink the material. (**b**) The resulting gel, shown in the vial, is immersed in buffer containing the reducing agent NADH and is exposed to CH_4_ and air. The resulting methanol and protein are quantified to determine the specific activity of the material.

A schematic of the method used to fabricate the PEG-pMMO hydrogels is shown in Fig. 1. The synthesis of the PEG-pMMO materials required only membrane-bound pMMO, PEGDA macromer, photoinitiator (not shown, see Methods) and ultraviolet light. We focused on PEGDA with a molecular weight of 575g mol^−1^ because shorter PEGDA lengths inactivated the enzyme and longer PEGDA lengths did not increase enzyme activity in initial studies (Supplementary Fig. 1). Since photoinitiator concentrations higher than 0.5 vol% in PEGDA decreased the pMMO activity, the photoinitiator concentration was held constant at 0.5 vol%. We included membrane-bound pMMO alone in each activity assay as a positive control. It should be noted that the membrane-bound pMMO preparations were crude fractions separated from cell material, and likely contained various other membrane-bound enzymes such as an NADH-quinone oxidoreductase that allows the use of NADH to supply electrons to pMMO^15^. The measured activities of this control were variable from experiment to experiment, 75–200 nmol MeOH mg^−1 ^min^−1^, while the values for the optimized PEG-pMMO samples were 65–128 nmol MeOH mg^−1^ min^−1^. The measured activities for both membrane-bound pMMO alone and immobilized pMMO are similar to the published values of 80–130 nmol MeOH mg^−1 ^min^−1^ for membrane-bound pMMO with methane as a substrate and NADH as a reductant^32^.

**Figure 2:**
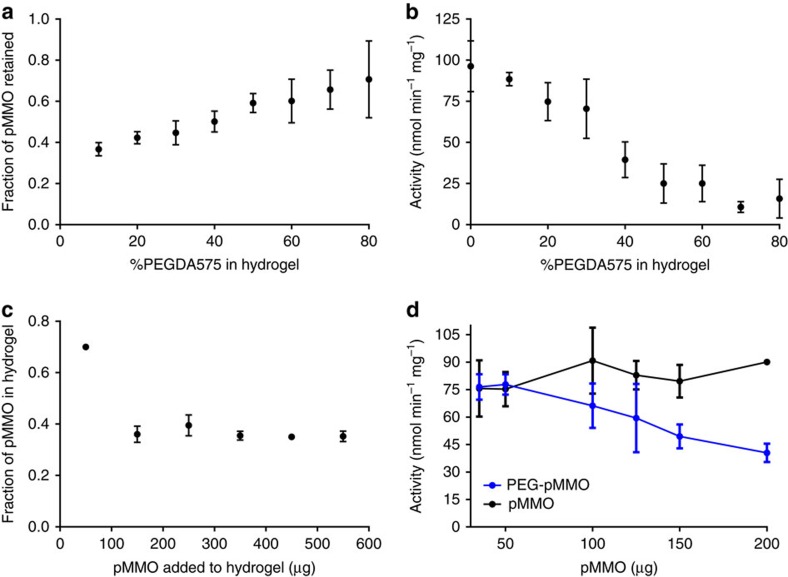
The effect of PEGDA and pMMO content on pMMO retention and activity. The effect of PEGDA percentage by volume during polymerization on (**a**) pMMO retention (by weight) and (**b**) enzyme activity when fabricated with 150 μg pMMO. (**c**) Amount of pMMO that is retained in the PEGDA 575 hydrogel as a function of the amount of pMMO included during polymerization. (**d**) Activity of PEG-pMMO and pMMO control as a function of pMMO (μg) included during the activity assay. Each experiment was repeated in triplicate with *N*=2 and error bars represent the s.d. across the set of three experiments.

The effects of systematically increasing the volume % of PEGDA in the solution before curing on protein retention and activity were then investigated (Fig. 2). Mixing the pMMO solution with PEGDA at the appropriate vol% (10–80%) followed by ultraviolet curing resulted in 50 μl PEG-pMMO hydrogels. As the PEGDA vol% was increased from 10 to 80%, the stiffness (observed during material handling) of the material increased and the amount of residual liquid on the surface of the hydrogel decreased. We observed a gradual increase in the fraction of pMMO that was retained (0.4–0.75) when the PEGDA vol% was increased from 10 to 80% (Fig. 2a).

However, a dramatic decrease in pMMO activity was observed as the PEGDA vol% was increased (Fig. 2b). At 10% PEGDA, the pMMO activity was ∼88±4 nmol MeOH min^−1^mg^ −1^, which closely corresponded to the activity of pMMO alone (96±15 nmol MeOH min^−1^mg^−1^; Fig. 2b). However, this value dropped below 30 nmol MeOH min^−1 ^mg^−1^ when the PEGDA vol% was greater than 50% (Fig. 2b). The amount of pMMO retained in the hydrogel before and after the activity assay did not change, indicating that no pMMO leached out during the activity assay and that the enzyme was entrapped efficiently in the hydrogel. Since only a marginal increase in pMMO retention (0.4 versus 0.42) and a more significant decrease in pMMO activity (88 versus 74 nmol MeOH min^−1 ^mg^−1^) was observed when the PEGDA vol% was increased from 10 to 20%, all remaining experiments were performed using 10 vol% PEGDA.

The concentration of pMMO used during hydrogel fabrication also affects retention and activity (Fig. 2c,d). For these experiments, the amount of pMMO used to generate the 50-μl PEG-pMMO hydrogel was varied between 50 and 550 μg. The fraction of pMMO retained was the highest at the lowest pMMO concentration tested (50–0.75 μg retained), and a dramatic decrease was observed when the amount of pMMO was increased to 150 μg (∼0.4 retained; Fig. 2c). Interestingly, we did not observe any further changes in the fraction of pMMO retained when the pMMO was increased up to 550 μg. To assess the effect of pMMO concentration in the PEG-pMMO hydrogel on activity, PEG-pMMO hydrogels were prepared with 50–550 μg of pMMO, which resulted in retention of 35–200 μg of pMMO in the hydrogel, and the activity was measured. The pMMO activity in the hydrogel was similar to the activity of pMMO alone when the amount of pMMO retained was below 50 μg; however, the activity in the hydrogels decreased gradually as the pMMO levels were increased from 50 to 200 μg. This effect was not observed in the pMMO alone sample (Fig. 2d).

There are several possible explanations for the diminished pMMO activity at increased polymer concentrations. Although it has been shown that PEDGA concentration (and by correlation, crosslinking density) has minimal effect on methane permeability in the gas phase^25^,^33^, gas permeability is affected by the hydration (swelling) of hydrogel materials^34^,^35^. Thus, PEGDA concentration may have an impact on methane permeability in swollen PEG-pMMO. Higher PEGDA concentrations also decrease the distance between crosslinks and the diffusion of aqueous solutes through the hydrogel^36^,^37^,^38^, and could limit diffusion of the NADH cofactor to the enzyme or diffusion of the methanol product from the active site. In order to test the effect of increasing the distance between crosslinks on enzyme activity, we compared the activity of pMMO in PEGDA 575 with PEGDA 2000 molecular weight (g mol^−1^) (which has roughly 3.5-fold greater distance between crosslinks). The results indicate a possible slight improvement in activity, from 82 to 97 nmol MeOH min^−1 ^mg^−1^ for the pMMO in PEGDA 2000; however, this increase is not statistically significant (Supplementary Fig. 1). In addition, the enzyme retention in the PEGDA 2000 was about half that in the PEGDA 575, justifying the focus on PEGDA 575 for this study. Moreover, the photo-initiated crosslinking reaction used to generate the crosslinked hydrogel results in the generation of deleterious free radicals^37^,^39^,^40^, which can oxidize amino acids^41^ and cleave peptide bonds^42^. A higher polymer content may be achievable by changing the curing chemistry in order to reduce the number of radicals generated. Our optimized PEG-pMMO formulations are remarkable in that they preserve physiological activity of pMMO in a tunable, ultraviolet-crosslinked polymeric material without harsh crosslinking conditions or organic solvents that could decrease enzyme activity, and furthermore the ability to crosslink by ultraviolet-initiated polymerization is highly amenable to 3D printing applications.

### Reuse and stability of PEG-pMMO

**Figure 3:**
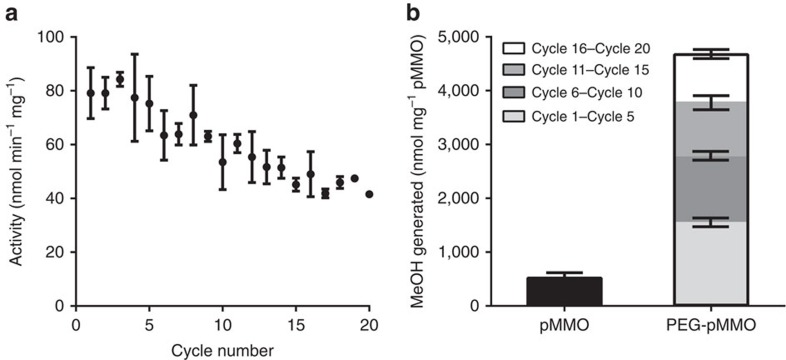
The effect of reusing the PEG-pMMO hydrogel over multiple cycles. (**a**) pMMO activity in the PEG-pMMO hydrogel during the course of 20 methane activity assay cycles. (**b**) Cumulative amount of methanol (nmoles) produced per mg of pMMO for both as-isolated membrane-bound pMMO and PEG-pMMO over 20 cycles of the methane activity assay. Error bars represent the s.d. from the average of four replicates.

The active pMMO-containing polymer material allowed facile reuse of pMMO. PEG-pMMO hydrogels prepared with 150 μg and 10 vol% pMMO were subjected to 20 cycles of 4-min exposures to methane (Fig. 3). The hydrogel was washed thoroughly between cycles to ensure that no residual methanol product remained in the hydrogel. We measured the protein content in the reaction buffer for each cycle to verify that the pMMO concentrations remained constant, and that there was no leaching through the course of the measurements. The activity between assay cycles 1 and 5 remained close to the initial activity (∼80 nmol MeOH min^−1 ^mg^−1^) and then gradually decreased to ∼45 nmol MeOH min^−1 ^mg^−1^ after 20 cycles (Fig. 3a). These 20 consecutive reactions of PEG-pMMO yielded 10-fold more methanol than that produced by a single reaction of membrane-bound pMMO (Fig. 3b) The average turnover number of the enzyme in PEG over 20 cycles is 3,921±879 nmol MeOH per mg of enzyme. Extrapolating the trend, the material appears to approach a lifetime turnover of 4,300±874 nmol MeOH per mg of enzyme. Although membrane-bound pMMO could be reused in principle, immobilization in the hydrogel renders reuse completely straightforward.

### Continuous methanol production using a flow-through bioreactor

**Figure 4:**
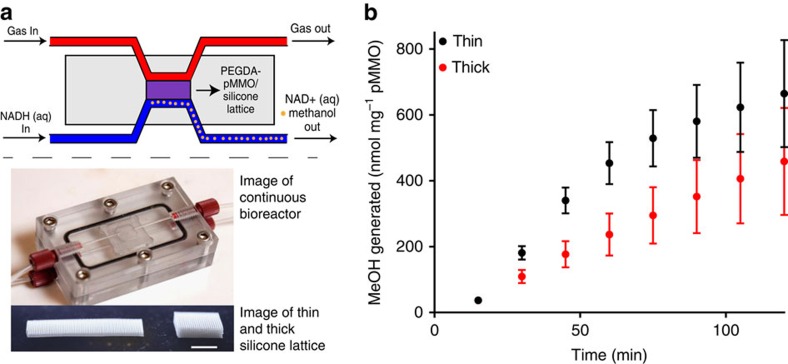
Continuous methanol production using a flow-through bioreactor design. (**a**) Schematic and image of the flow-through bioreactor and the two (thin and thick) silicone lattice structures used to support the PEG-pMMO hydrogel membrane (scale bar, 1 cm). (**b**) Amount of methanol (nmole) produced per mg of pMMO in the PEG-pMMO hydrogel bioreactor showing continuous MeOH production for 2 h in thin versus thick membrane at 45 °C. Error bars represent the s.d. of three samples.

The successful recycling of the PEG-pMMO material opened the possibility of its use in a bench-scale continuous flow reactor. The fact that pMMO acts upon gas phase reactants and generates liquid phase products suggests a design in which the pMMO material is suspended between gas and liquid reservoirs. However, PEG-pMMO, and hydrogels in general, are mechanically brittle and difficult to handle when molded as thin membranes. To increase the mechanical stability, we embedded the PEG-pMMO material into a 3D silicone lattice (printed using Direct Ink Write), which also ensures easy tunability of the hydrogel's size and shape. The lattice was constructed of 250-μm silicone struts and contained 250-μm void spaces (50% porosity), which were infilled with PEGDA 575, pMMO membrane preparations and photoinitiator and then crosslinked in place with ultraviolet light. Two such lattice structures, thin and thick with surface area to volume ratios of 5 to 1, were designed to compare effects of PEG-pMMO surface area to volume ratio on methanol production (Fig. 4a). The silicone lattice structure increases the bulk gas permeability of the material, as silicone permeability is at least 50 times greater than that of PEGDA hydrogel^25^,^43^.

The resulting hybrid silicone-PEG-pMMO lattice materials were mechanically robust, and could be suspended at 1-mm thickness between gas and liquid reservoirs in a flow-through reactor (Fig. 4a). With this configuration, we could flow a methane/air gas mixture on one side of the lattice and introduce the NADH on the other side while continuously removing and collecting methanol in buffer. To test whether the initial NADH concentration was sufficient in the flow experiment, we tested the enzyme activity as a function of NADH concentration in the supernatant in batch tests, where PEG-pMMO was soaked in various NADH concentrations for 5 min before measuring the activity (Supplementary Fig. 2). We did not see any statistically significant change in the enzyme activity even at 1/8 the concentration used in the flow experiment (0.75 versus 6 mM). Because PEGDA material was pre-soaked in NADH for 10 min before the start of the flow experiments, these data indicate that (1) we used an excess of NADH in the flow experiments and (2) the PEGDA-pMMO was at the equilibrium NADH concentration at the start of the experiment. To determine the length of time the membrane could be used continuously, we measured the cumulative methanol produced per mg of enzyme at 25 °C at 30-min intervals in the thick lattice over the course of 5.5 h (Supplementary Fig. 3). The methanol production rate (slope of methanol versus time curve) was stable for ∼2.5 h and declined gradually over the next 3 h. To evaluate whether the geometry of PEG-pMMO material influenced methanol production rates, reactor outlet fractions from reactors containing the thin and thick lattices were compared at 15-min intervals at 45 °C over the course of 2 h (Fig. 4b). The methanol produced (per mg of protein) by the thin membrane (black squares) was double that produced by the thick membrane (red circles) over the course of the first hour, but then declined relative to that of the thick membrane; after 2 h the average total methanol produced by the thin membrane was 1.5 times higher than that produced by the thick membrane. The methanol concentrations produced in the flow reactor were on average 12 and 6% of those predicted for thin and thick lattices, respectively, based on analyte flow rates and an assumed pMMO activity of 80 nmol MeOH min^−1 ^mg^−1^. The low methanol concentration values relative to predicted values may be because of lower actual pMMO concentrations in the material than was calculated based on dilution from the stock of known concentration. In addition, a small amount of methanol (20 μM) was detected in the liquid through which the gas was bubbling, suggesting that a fraction of the methanol generated was lost as vapour to the gas stream and not collected in the liquid fractions. Further reactor development is needed to efficiently capture both gas and liquid phase methanol.

A full understanding of the mass transport and reaction characteristics of this system would require a kinetic model of the enzyme, which is not yet available, and a finite element model of the membrane, which is beyond the scope of this paper. We have, however, made some rough calculations to assess which species may be limiting, described in detail in Supplementary Note 1. The modelled mass transfer rates are listed in Supplementary Table 1. Considering the ratios of the characteristic flux for reaction and diffusion, we find that the thick membrane is likely mass transfer-limited in both O_2_ and NADH. The thin membrane may be reaction-limited only, unless the crosslinking of the PEG significantly slows the transport of NADH. In that case the thin membrane may also be NADH-limited, but less so. In either case, the calculations suggest that a polydimethyl siloxane (PDMS) matrix like the ones prototyped can supply gas phase reactants at sufficient rates to maximize enzyme activity through a 1–3-mm membrane.

### Direct printing of PEG-pMMO hydrogel

**Figure 5:**
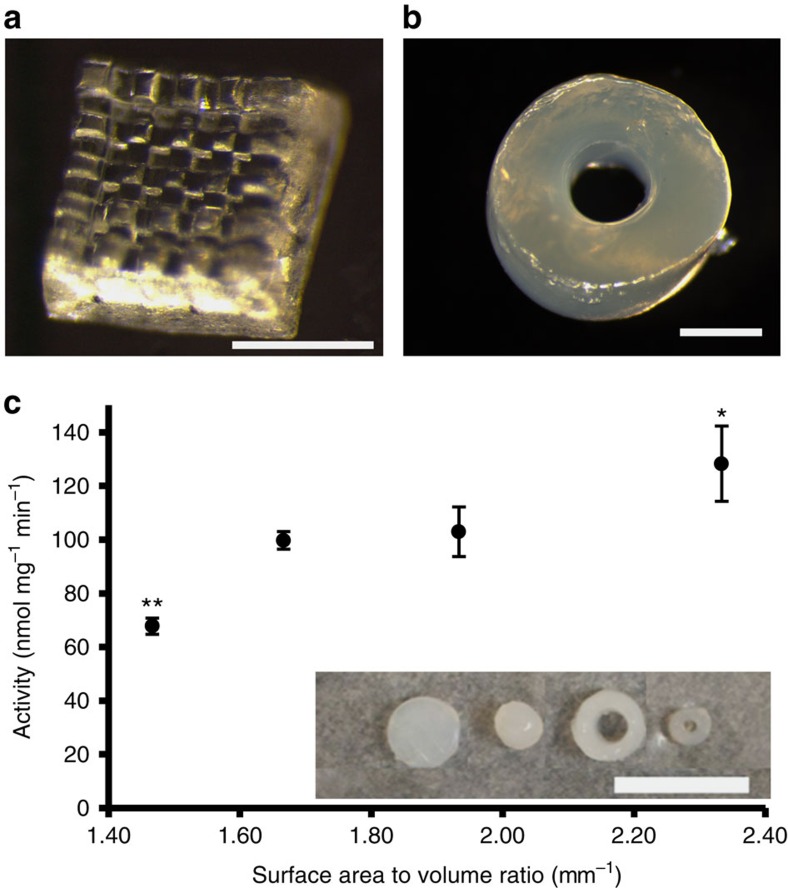
PEG-pMMO structures printed using PμSL. Printing PEG-pMMO structures with PμSL allows high-resolution (features on the order of tens of μm) and flexibility in bioreactor component design. (**a**) Printed PEG-pMMO grid structure with small feature size (scale bar, 500 μm). (**b**) Large area PμSL was used to print cylinders with varying surface area to volume ratios on a shorter timescale with reduced resolution (scale bar, 1 mm). (**c**) The dependence of PEG-pMMO activity on surface area to volume ratio (*N*=3, error bars represent s.d., statistical significance determined by pairwise *t*-test where *P<0.1, **P<0.05). Inset: printed cylinders with surface area to volume ratios of (left to right) 1.47, 1.67, 1.93 and 2.33 (scale bar, 10 mm).

Since the ability to tune the shape and proportions of immobilized pMMO had an impact on the performance of the biocatalytic material even at the millimetre scale, we next investigated the effects of using new methods of printing polymer architectures with resolution on the 10–100-μm scale. Projection microstereolithography (PμSL) allows 3D printing of light-curable materials by projecting a series of images on the material, followed by changing the height of the stage in discrete increments, with micron-scale resolution in all three dimensions^44^. Therefore, it is an ideal technique for directly printing the PEG-pMMO material and determining whether changing geometrical features of the material at these length scales can influence activity. We used PμSL to print PEG-pMMO lattice structures with increased surface area to volume ratio conferred by 100-μm^2^ vertical hollow channels in a 1,000-μm^2^ hydrogel block with a height of ∼500 μm, corresponding to ∼85% hydrogel volume and ∼15% void (empty) volume (Fig. 5a). The vertical channels were inserted to increase the surface area of the structure and thereby increase mass transfer into the hydrogel. In this proof-of-concept experiment, the pMMO concentration of 5 mg ml^−1^ did not attenuate the light enough for highest-resolution printing; consequently, feature resolution was reduced in the *z* direction and each layer of printed pMMO was exposed to multiple exposures to ultraviolet light. The pMMO activity in the printed lattices with a total volume of ∼27 mm^3^, which took ∼50 min to print using PμSL, was reproducibly 29 nmol MeOH min^−1 ^mg^−1^. The reduction in activity compared with membrane-bound pMMO is likely because of the duration of the printing at room temperature as well as the overexposure of pMMO to ultraviolet light during curing. However, the cubic lattices retained ∼85% of the enzyme based on the solid volume of the lattice (23 mm^3^) corresponding to the highest protein loading that we have achieved. This high retention is likely because of increased crosslinking efficiency.

Since the lattice geometry did not permit precise tuning of surface area to volume ratios due to bending of lattice struts under water surface tension, a different PμSL tool designed to generate larger parts was used to print solid and hollow PEG-pMMO cylinders with surface area to volume ratios of 1.47–2.33 and diameters of 1–5 mm (Fig. 5b). We hypothesized that the hollow tube geometry would allow more facile diffusion of reactants because both the inner and outer surfaces of the cylindrical materials would be exposed. The total print time for an array of cylinders using the large-area PμSL (LAPμSL) tool was significantly reduced to ∼1 min by eliminating *z* axis resolution, and the pMMO concentration was reduced to 2.3 mg ml^−1^ to allow ultraviolet light penetration through the 1.5–3-mm depth of the resin. Remarkably, the activity of pMMO in the hydrogels increased with greater surface area to volume ratios (Fig. 5c), with the highest ratio of 2.33 resulting in an average activity of 128±14 nmol MeOH min^−1 ^mg^−1^ per cylinder, which corresponds to the maximum reported activity of membrane-bound pMMO with NADH as a reducing agent^32^. The cylinders of the lowest ratio, 1.47, had an average pMMO activity of 67±3 nmol MeOH min^−1 ^mg^−1^. It should also be noted that the cylinders with the lowest surface area to volume ratio were only 1.5 mm in height and therefore completely submerged in the liquid phase during the activity assay, whereas all other cylinders tested were 3 mm in height and only partially submerged during the assay. Hydrogels protruding from the liquid allow a direct interface between the gas phase and PEG-pMMO, and this exposed interface likely increased the methane concentration in the PEG-pMMO material^26^. On average, 38% of the protein was encapsulated, although it was variable depending on the dimensions of each cylinder (27–54%).

These results, combined with the results from the continuous flow reactor, indicate that an optimal pMMO membrane design might have a range of feature sizes depending on pMMO loading and reaction conditions, with the smallest feature sizes on the order of hundreds of microns. It appears that, because of the kinetics of pMMO, thinner membranes yield higher activity up to a threshold where the activity reaches a maximum. The optimal membrane thickness should be thin enough to maximize pMMO activity without making the reactor unnecessarily complex or fragile, and consequently depends on the pMMO loading in the polymer and the reactor configuration. Figure 5c suggests that a feature thickness of up to 1 mm is sufficient to optimize mass transfer in PEG-only membrane at those experimental conditions, whereas our calculations in Supplementary Note 1 for the flow-through reactor suggest that a composite membrane around 1–3 mm would be sufficiently thin, given the conditions of that experiment.

## Discussion

A number of advancements must be made to realize a deployable MMO-based bioreactor for reducing methane emissions from remote locations. These include process intensification to address kinetic limitations related to gas mass transfer, increasing the native MMO stability through protein engineering, the economical separation of pure methanol from the aqueous reactant mixture and the economical supply of reducing agent. This work focuses on immobilizing pMMO and improving mass transfer in the key methane activation step. We have developed an enzyme–polymer hybrid material consisting of active pMMO and associated lipids entrapped within a PEG-based hydrogel. Enzyme immobilization within a polymer has been employed for biosensors^45^, enzymatic fuel cells^46^ and drug delivery^47^. The use of a ultraviolet-curable polymer for the immobilization of a membrane-bound enzyme with 100% retention of activity, and 3D printing of a biocatalytic material for improving mass transfer is unique. Our strategy of ultraviolet-crosslinking pMMO-containing lipid membranes in a polymeric material (1) allowed pMMO to retain its native conformation, preserving the physiological activity, (2) allowed reuse and continuous use of pMMO in flow-through bioreactor prototype devices and (3) allowed facile printing of the active pMMO into 3D structures with tunable geometries from micron to millimetre scales. An additional advantage to immobilizing pMMO within a polymer, rather than on the surface of an impermeable support, is the potential to fully embed pMMO throughout the depth of the support material for high loading. Embedding a cell membrane preparation from a methanotroph within a crosslinked hydrogel thus provides a simple and inexpensive route for maintaining the activity of a membrane enzyme in an immobilized form. Moreover, the ability to print and alter the geometry and surface area of the biocatalytic material affects the activity, underscoring the general importance of the printed bioreactor concept.

Our results also demonstrate that biocatalytic polymers are readily employed as ‘inks' for 3D printing. Advances in 3D printing of biological materials have been almost exclusively focused on biomedical applications^48^ because biological tissues and organs have a highly complex hierarchical 3D structure, which can only be replicated using 3D bioprinting^49^. A bioreactor that optimizes mass transfer, diffusion-limited and kinetic-limited reactions is similarly complex, and precise control over the 3D spatial distribution of enzymes is beneficial in optimizing product production. The ability to print multiple biocatalytic materials in close proximity with micron-scale features may also facilitate more complex mimics of natural biocompartmentalization, allowing the immobilization of synthetic pathways and cofactor regeneration. The spatial organization of enzymes in living organisms enables substrate channelling by decreasing diffusion distances between complementary enzyme containing materials, reducing the probability of unfavourable synthetic pathways and isolating unfavourable products^50^,^51^.

To make process improvements to large-scale methanol production, 3D printing could be used to directly fabricate reactor components, or to prototype reactor component geometries, which might later be fabricated as enzyme-embedded polymer components using conventional means (for example, molding or extrusion). 3D printing allows fabrication of geometries that are inaccessible by conventional means, the ability to print enzyme gradients (to minimize the amount of enzyme required for a given volumetric productivity) and the ability to control the location of multiple materials within a structure (for example, to supply reducing equivalents). Ultimately, the cost of 3D printing the reactor components would need to be balanced with the process improvements enabled by the 3D printing.

This work opens a pathway for high-throughput biocatalysis suitable to the large-scale and gas–liquid reactions in the energy sector. However, several important hurdles remain, such as achieving enzyme longevity, enhancing throughput and situating pMMO within a larger reaction chain that supplies the necessary reducing agent. NADH is costly, unstable and not viable for industrial use. A convenient reductant for industrial use would be hydrogen, which can be used to regenerate NADH catalysed by hydrogenase enzymes, and which can be reformed from a portion of the methane fed to the system. Such a reaction might be achieved by co-immobilization of hydrogenase^52^ with pMMO, which would have the further advantage that all the reactants are gas phase and all the products are aqueous, simplifying reactant–product separation. Other options include supplying the electrons electrochemically, or co-immobilizing pMMO with methanol dehydrogenase to yield formaldehyde as the product for collection. With these challenges in mind, the application of biological tools to address the need to convert fugitive and remote methane streams requires that biologists and materials scientists work together to optimize biocatalysts through protein engineering and to develop accompanying bioreactor materials.

## Methods

### Materials

Reagents for buffers (PIPES, NaCl and NaOH), HPLC-grade methanol (≥99.9% purity), NADH, PEGDA 575 and the crosslinking initiator, 2-hydroxy-2-methylpropiophenone (Irgacure 1173) were purchased from Sigma-Aldrich (St Louis, MO). All reagents were used as received. Methane gas (99.9% purity) was obtained from Matheson Tri-gas Inc. (Basking Ridge, NJ). Lithium phenyl-2,4,6-trimethylbenzoylphosphinate photoinitiator was synthesized following the procedure set forth by Majima *et al.*^53^.

### Cell growth and membrane isolation

*M. capsulatus* (Bath) cells were grown in 12–15 l fermentations as previously described^32^,^54^. *M. capsulatus* (Bath) cells were grown in nitrate mineral salt medium (0.2% w/v KNO_3_, 0.1% w/v MgSO_4_·7H_2_O and 0.001% w/v CaCl_2_·2H_2_O) and 3.9 mM phosphate buffer, pH 6.8, supplemented with 50 μM CuSO_4_·5H_2_O, 80 μM NaFe(III)EDTA, 1 μM Na_2_MoO_4_·2H_2_O and trace metal solution. Cells were cultured with a 4:1 air/methane ratio at 45 °C and 300 r.p.m. Cells were harvested when the A_600_ reached 5.0–8.0 by centrifugation at 5,000*g* for 10 min. Cells were then washed once with 25 mM PIPES, pH 6.8 before freezing in liquid nitrogen and storing at −80 °C. Frozen cell pellets were thawed in 25 mM PIPES, pH 7.2, 250 mM NaCl buffer (herein referred to as pMMO buffer) and lysed by microfluidizer at a constant pressure of 180 p.s.i. Cell debris was then removed by centrifugation at 20,000–24,000*g* for 1 h. The membrane fraction was pelleted by centrifugation at 125,000*g* for 1 h and washed three times with pMMO buffer before freezing in liquid nitrogen and storing at −80 °C. Final protein concentrations were measured using the Bio-Rad DC assay. Typical storage concentrations ranged from 20 to 35 mg ml^−1^.

### Preparation of the PEG-pMMO hydrogels

Before preparation of the PEG-pMMO hydrogels, frozen as-isolated membranes from *M. capsulatus* (Bath; herein referred to as membrane-bound pMMO) were thawed at room temperature and used within 5 h of thawing. Thawed membrane-bound pMMO (50–500 μg) was then mixed with PEGDA 575 in pMMO buffer at room temperature to a final volume of 50 μl and 10–80 (v/v %) PEGDA 575. Initiator (2-hydroxy-2-methylpropiophenone) was included at 0.5 vol% with respect to PEGDA 575, unless stated otherwise. The solutions were mixed by pipetting to homogeneity and then transferred to a 1-ml syringe with the tip removed. The syringe was then immediately placed under ultraviolet light at 365 nm, 2.5 mW cm^−2^ intensity, for 3 min. After the ultraviolet exposure, the 50 μl PEG-pMMO hydrogel block was slowly pushed out of the syringe on a kimwipe where it was gently blotted and then rinsed twice in pMMO buffer to remove unreacted reagents. We used fluorescently labelled liposomes as a surrogate for pMMO liposomes/crude lipid extracts in order to image the distribution of liposomes in the PEG hydrogels. The results indicated uniform distribution at the resolution of the confocal microscope, 500 nm. The images and description of the experiment are shown in Supplementary Fig. 4.

### Activity assay

pMMO activity was measured as previously described^32^,^55^ with slight modifications. Briefly, all reactions were carried out in 2 ml glass reaction vials in pMMO buffer with 6 mM NADH as a reducing agent. Vials with 50–500 μg pMMO in 125 μl buffer solution were used as controls. For the immobilized enzyme samples, each 50 μl PEG-pMMO hydrogel block was placed in a vial and partially submerged in 75 μl buffer solution immediately after curing and rinsing. Headspace gas (1 ml) was removed from each vial using a 2-ml gas-tight glass syringe and replaced with 1 ml of methane, and the reaction vial was immediately placed in a heating block set at 45 °C and incubated for 4 min at 200 r.p.m. After 4 min, the samples were heat-inactivated at 80 °C for 10 min. Samples were then cooled on ice for 20 min, and pMMO control vials were centrifuged to remove the insoluble membrane fraction. For the cyclic activity assays using the PEG-pMMO immobilized enzyme, the reaction was stopped by opening and degassing the head space and immediately removing the solution for GC analysis. The block was then rinsed three times with 1 ml of pMMO buffer per wash and the assay was repeated. The amount of methanol generated during the reaction was measured by gas chromatography/mass spectrometry (GC/MS) analysis using an Agilent Pora-PLOT Q column, and calibration curves were generated from methanol standards. Typical background signals due to methanol contamination given as a percentage of the positive control signal were <1% from the pMMO buffer, <1% from PEGDA and <2% from NADH.

### pMMO flow reactor

A simple cubic PDMS lattice with 250-μm struts and 250-μm spacing was printed using Direct Ink Write as described^56^ to provide methane permeability throughout the PEG material and to provide mechanical support. A top layer of 50-μm-thick PDMS was fabricated by spin-coating Dow Corning SE-1700 PDMS diluted in toluene on a hydrophobized silicon wafer. This thin PDMS membrane prevented leakage of liquid through the membrane but provided gas permeability. Two different flow cell geometries were fabricated using polycarbonate plastic: a flow cell for a higher surface area, thin lattice (1.25 cm wide by 3 cm long) and a lower surface area, thick lattice (1.25 by 1.25 cm). The thin lattice was six-layers thick, and the thick lattice had 16 layers. The lattices were made hydrophilic by treating them in air plasma for 5 min, followed by storage in deionized water. To incorporate the pMMO into the lattices, a 10 vol% concentration of PEGDA 575 was mixed with membrane-bound pMMO to a final concentration of 5 mg ml^−1^ pMMO. The pMMO/PEGDA mixture (200 μl) was pipetted into the lattice and cured with 365 nm ultraviolet light at 2.5 mW cm^−2^ intensity for 4 min, forming the mixed polymer (PEG/PDMS) membrane. The final concentration of pMMO in the lattices was calculated, rather than directly quantified using a protein assay because of difficulties in quantifying the material in the lattice. The membrane was then loaded into the cell and rinsed with buffer to remove any unpolymerized material.

The flow cell was placed on a hot plate calibrated with thermocouple so that the membrane would reach either 25 or 45 °C. An NADH/buffer solution (4 mg ml^−1^ NADH in PIPES pH 7.2) was prepared as the liquid phase in a 5-ml syringe, and the gas phase was prepared as 50% methane and 50% air loaded into a gas-tight 50-ml syringe. The syringes were loaded into Harvard Apparatus syringe pumps and the gas and liquid were delivered at 0.5 and 0.75 ml h^−1^, respectively. The gas outlet tubing was kept under 2-cm water pressure during the reaction. Fractions of liquid were collected into GC/MS autosampler vials that were kept on ice to reduce methanol evaporation and were analysed against methanol standards using GC/MS as described above. Methanol contamination was present in the NADH/buffer solutions, and this concentration was subtracted from the total detected in each fraction by GC/MS. No methanol contamination was found in the water used to store the PDMS. The data shown in Fig. 4b represent cumulative methanol production in which the quantity of methanol produced in each fraction was added to the previous samples. Each experiment was performed in triplicate; the error bars represent a s.d.

### 3D printing of PEG-pMMO hydrogels

The printing resin is comprised of 20 vol% PEGDA 575, 10 mg ml^−1^ lithium phenyl-2,4,6-trimethylbenzoylphosphinate initiator and 2.3–5 mg ml^−1^ crude pMMO in buffer. Using PμSL, hydrogel blocks were printed in a cubic lattice with 100-μm open channels spaced 100 μm apart and size dimensions of 1–3 mm. Solid and hollow cylinders of the same resin formulation were printed using the LA PμSL system. The cylinders had an inner diameter of 1–2.5 mm, an outer diameter of 3–5 mm and were 1.5–3 mm tall. The resin was cured with a 395-nm diode with both PμSL and LA PμSL but the intensity and exposure time varied between the systems, ranging from 11.3 to 20 W cm^−2^ and 15–30 s per layer. Resin and printed hydrogels were stored on ice before and after the printing process. The pMMO activity assay was carried out as described above at 45 °C for 4 min, and the methanol and protein concentrations of the printed hydrogels were measured as described above.

### Data availability

The data that support the findings of this study are available from the corresponding author (S.E.B.) upon request.

## Additional information

**How to cite this article:** Blanchette, C. D. *et al.* Printable enzyme-embedded materials for methane to methanol conversion. *Nat. Commun.* 7:11900 doi: 10.1038/ncomms11900 (2016).

## Supplementary Material

Supplementary Information Supplementary Figures 1-4, Supplementary Table 1, Supplementary Note 1 and Supplementary References
