# Supplementary material for: Printable enzyme-embedded materials for methane to methanol conversion
Source: Nat Commun. 2016 Jun 15;7:11900. doi: 10.1038/ncomms11900 (PMC4912616; doi:10.1038/ncomms11900)
Supplement: Supplementary Information — Supplementary Figures 1-4, Supplementary Table 1, Supplementary Note 1 and Supplementary References [file ncomms11900-s1.pdf]

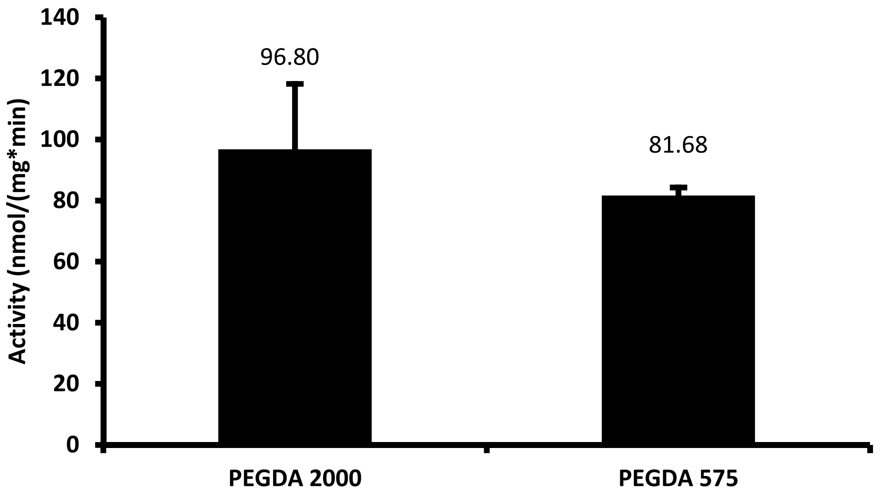

Supplementary Figure 1. Effect of PEG length on pMMO activity. Enzyme activity in PEGDA2000 or PEGDA575 hydrogels as determined by a methanol assay. Values were not significantly different according to a Student's T-test.

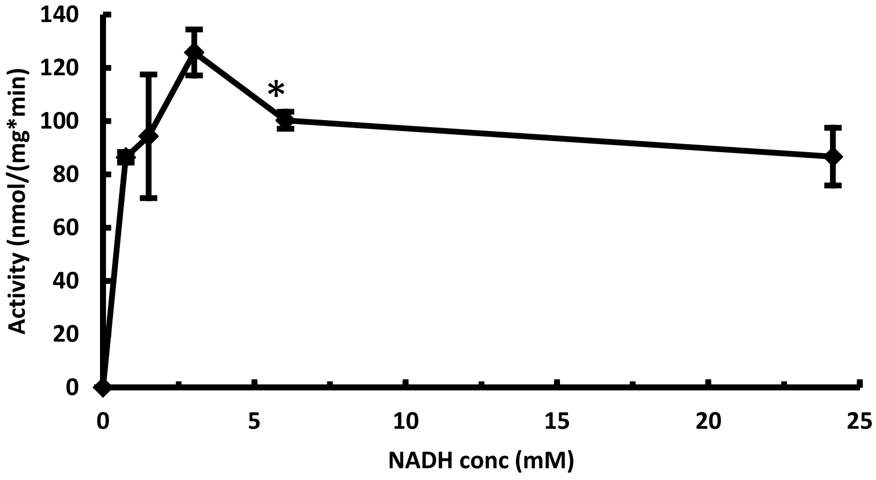

Supplementary Figure 2. Effect of NADH concentration on pMMO activity. Enzyme activity as determined by a methanol assay in PEG-pMMO gels prepared with 10 vol% PEGDA575 and 150 μg pMMO as a function of NADH cofactor concentration of 0.75, 1.5, 3, 6, and 24 mM (no statistical significance according to ANOVA analysis). *Concentration used in typical activity assays.

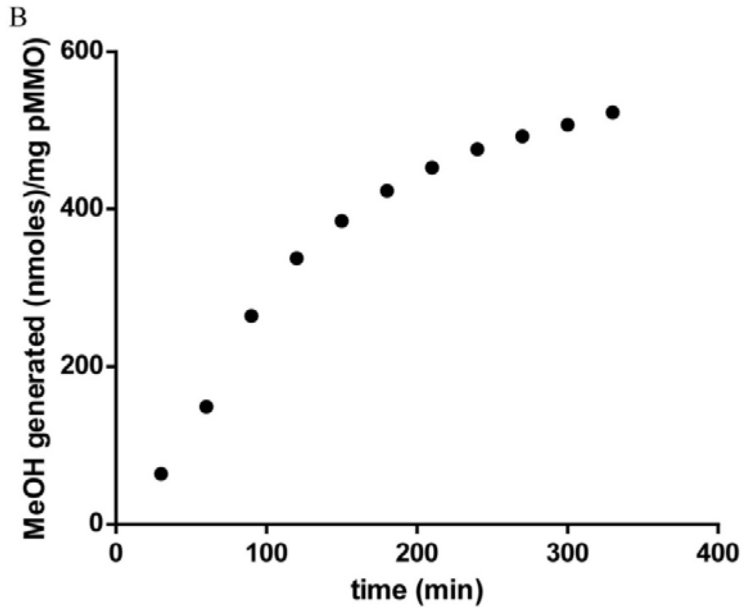

Supplementary Figure 3. Continuous methanol generation in flow reactor. Methanol produced (cumulative) in flow-through reactor at 25 °C.

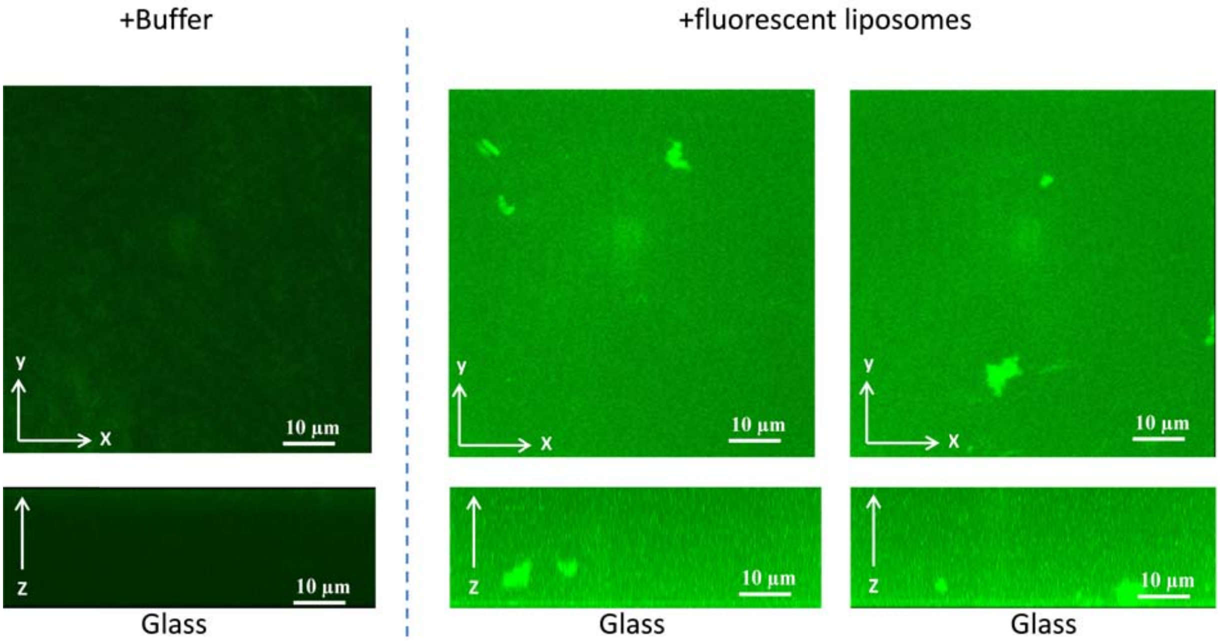

Supplementary Figure 4. Liposome distribution within PEG hydrogels. Confocal fluorescence microscopy images of fluorescently labeled liposomes in 10 vol% PEGDA 575. Liposomes were a mixture of 99 mol% DOPC and 1 mol% 12:0 NBD PC (Avanti Polar Lipids). Liposomes were prepared by drying a lipid film at the indicated molar ratios and rehydrating in PBS to a final concentration of 5 mg/ml. The liposome suspension was then extruded through a 100 nm pore to generate 100 nm diameter liposomes. The extruded liposome suspension was then mixed with 10% PEGDA 575 and crosslinked as described for the pMMO suspension. The resulting hydrogel was sandwiched between two glass cover slips for imaging. Images were acquired using a Z confocal microscope at excitation and emission wavelengths of 470 nm and 540 nm, respectively. The images show mostly uniform fluorescence with some brighter particles, likely scattering from dust. The image resolution is 500 nm; the images indicate uniform distribution of liposomes, a surrogate for the pMMO lipids, in crosslinked PEG over the scale of the images (scale bar = 10 μm).

	Permeability [m^2 s^{-1}]	Feed conc. [mol m^{-3}]	Thick membrane: Characteristic flux, diffusion [mol m^{-2} s^{-1}]	Thick membrane: Characteristic Flux, reaction [mol m^{-2} s^{-1}]	Thick membrane: Ratio rxn/diff	Thin Membrane: Characteristic flux, diffusion [mol m^{-2} s^{-1}]	Thin Membrane: Characteristic Flux, reaction [mol m^{-2} s^{-1}]	Thin Membrane: Ratio rxn/diff
CH4	1.10E-09	19.8	3.54E-06	2.42E-06	0.7	1.63E-05	4.05E-07	0.02
O2	6.90E-10	4.4	4.88E-07	2.42E-06	5.0	2.24E-06	4.05E-07	0.18
NADH	6.70E-10	6.0	4.96E-07	2.42E-06	4.9	1.02E-06	4.05E-07	0.40

Supplementary Table 1. Modeled characteristic mass transfer rates for reactants in the flow-through reactor. Ratios indicate whether mass transport is expected to limit overall activity, based on Equations 1-3.

Supplementary Note 1. Modeling reactant diffusion and reaction flux in PEG-PDMS lattices.

The reactive membrane in the flow-through apparatus consists of two phases: A PDMS phase printed as a regular mesh lattice of either 3.5 mm or 1 mm thickness, and a PEG hydrogel phase containing the enzyme. The PEG phase fills of the voids of the PDMS phase, creating two interconnected, porous networks. The membrane is designed to allow methane and oxygen to pass through the highly-permeable PDMS phase. The PEG phase provides a stable environment for the enzyme. Being polar and water-soluble, NADH and methanol most likely pass through the PEG phase primarily.

A full understanding of the mass transport and reaction characteristics of this system would require a kinetic model of the enzyme, which is not yet available, and a finite element model of the membrane, which is beyond the scope of this paper. We can, however, make some rough calculations to assess which species may be limiting.

Let us consider the relative rates of diffusion and reaction. We define the “characteristic flux” for diffusion, J_{diff} , as the maximum rate that a species would transport through the membrane without reaction. The characteristic flux for reaction, J_{rxn}, is the rate that reactants would be consumed if there was no resistance to mass transfer. If $J_{\mathrm{rxn}} \gg J_{\mathrm{diff}}$ or if they are on the same order for a species, we would expect some mass transfer limitation for that species. For methane and oxygen transport, we modify the basic formula for flux through a membrane to adjust for porosity:

$$J_{\mathrm{diff,gas}} = \frac{PC_{\mathrm{gas}}(1-\sigma)}{L} \quad (1)$$

where P is the permeability of the PDMS phase, C_{gas} is the concentration of gas above the membrane, σ is the volume fraction of PEG in the membrane, and L is the thickness of the membrane. For NADH transport, the analogous formula is:

$$J_{\mathrm{diff,NADH}} = \frac{PC_{\mathrm{NADH}}\sigma}{L} \quad (2)$$

where P is now the permeability of the PEG phase to NADH, is the volume fraction of the PEG phase. Because the stoichiometry for all three reactants is 1:1, the expression for J_{rxn} is the same for each. We suppose the enzyme is operating at its typical activity in dissolved form, then the flux to keep the membrane supplied with reactant is:

$$J_{\mathrm{rxn}} = k_0 m L \sigma \qquad (3)$$

where k_0 is the zero-order enzyme rate (activity) per mass of enzyme, and m is the mass loading of enzyme in the PEG phase.

The permeability of PDMS to CH_4 and O_2 are taken from the literature[1,2]. The permeability of the PEG phase to NADH is not known, but since the PEG phase is ~89% water, we suppose that the permeability is similar to the diffusivity of NADH in water, which is given by Wu[3]. Our experience with batch experiments supports the assumption that NADH transport in PEG is relatively fast on the 100—500 μm length scale (see '**Continuous methanol production using a flow-through bioreactor**' of the main text).

Results are shown in Supplementary Table 1. Considering the ratios of characteristic flux for reaction and diffusion, we see that the thick membrane is likely mass transfer-limited in both O_2 and NADH. The thin membrane may be reaction limited only, unless the cross-linking of the PEG significantly slows the transport of NADH. In that case the thin membrane may also be NADH-limited, but less so. In either case, the calculations suggest that a PDMS matrix like the ones prototyped can supply gas phase reactants at sufficient rates to maximize enzyme activity through a 1—3 mm membrane.

Supplementary References

1. Raharjo, R. D., Freeman, B. D., Paul, D. R., Sarti, G. C. & Sanders, E. S. Pure and mixed gas CH_4 and n-C_4H_{10} permeability and diffusivity in poly(dimethylsiloxane). *J. Membr. Sci.* **306,** 75–92 (2007).
2. De Bo, I. Investigation of the permeability and selectivity of gases and volatile organic compounds for polydimethylsiloxane membranes. *J. Membr. Sci.* **215,** 303–319 (2003).
3. Wu, Z., Jing, W. & Wang, E. Oxidation of NADH by dopamine incorporated in lipid film cast onto a glassy carbon electrode. *Electrochem. Commun.* **1,** 545–549 (1999).
